# Effects of α-Lipoic Acid Supplementation on Growth Performance, Liver Histology, Antioxidant and Related Genes Expression of Hybrid Grouper (*Epinephelus fuscoguttatus* ♀ × *E. lanceolatus* ♂)

**DOI:** 10.3390/antiox13010088

**Published:** 2024-01-10

**Authors:** Weibin Huang, Tao Li, Wenshan Cai, Hengyang Song, Hao Liu, Beiping Tan, Shuang Zhang, Menglong Zhou, Yuanzhi Yang, Xiaohui Dong

**Affiliations:** 1 Laboratory of Aquatic Nutrition and Feed, College of Fisheries, Guangdong Ocean University, Zhanjiang 524088, China; huangweibin31@stu.gdou.edu.cn (W.H.);; 2 Guangdong Engineering Technology Research Center of Aquatic Animals Precision Nutrition and High Efficiency Feed, Zhanjiang 524088, China; 3 Key Laboratory of Aquatic, Livestock and Poultry Feed Science and Technology in South China, Ministry of Agriculture, Zhanjiang 524000, China

**Keywords:** high-lipid diet, hybrid grouper, antioxidant, immunity, α-lipoic acid

## Abstract

This study aimed to assess the impact of α-lipoic acid on the growth performance, antioxidant capacity and immunity in hybrid groupers (♀ *Epinephelus fuscoguttatus* × ♂ *E. lanceolatus*) fed with a high-lipid diet. Groupers (8.97 ± 0.01 g) were fed six different diets, with α-lipoic acid content in diets being 0, 400, 800, 1200, 1600, and 2000 mg/kg, named S1, S2, S3, S4, S5, and S6, respectively. The results show that the addition of 2000 mg/kg α-lipoic acid in the diet inhibited the growth, weight gain rate (WGR), and specific growth rate (SGR), which were significantly lower than other groups. In serum, catalase (CAT) and superoxide dismutase (SOD) were significantly higher in the S5 group than in the S1 group. In the liver, CAT, SOD and total antioxidative capacity (T-AOC) levels were significantly increased in α-lipoic acid supplemented groups. α-lipoic acid significantly upregulated liver antioxidant genes *sod* and *cat*, anti-inflammatory factor interleukin 10 (*il10*) and transforming growth factor β (*tgfβ*) mRNA levels. Conclusion: the addition of 2000 mg/kg of α-lipoic acid inhibits the growth of hybrid groupers. In addition, 400–800 mg/kg α-lipoic acid contents improve the antioxidant capacity of groupers and have a protective effect against high-lipid-diet-induced liver oxidative damage.

## 1. Introduction

The world’s population has been increasing in recent years, leading to an increased demand for aquatic products [1]. To meet this demand, high-density intensive aquaculture has been widely promoted [2]. In intensive aquaculture, there is a growing preference for a high-lipid diet [3]. Lipids, along with other essential nutrients such as protein, carbohydrates and trace elements, are key components of aquatic animal feed [3]. Lipids are crucial components of fish tissue cells, and they can also be broken down to provide energy for the body’s self-repair [4]. Simultaneously, lipids serve as carriers for fat-soluble vitamins, promoting their absorption and positively impacting the body [5,6]. Against the backdrop of declining fishery resources nationwide [7], resulting in a consistent year-on-year decrease in fishmeal production and a rise in prices, the increase in raw material prices inevitably leads to a hike in feed prices. These consequences are severe: escalating farming costs for aquaculture practitioners, raising the aquaculture industry’s threshold and causing widespread unemployment, adversely affecting the global economy’s development. As researchers, we are constantly exploring methods to reduce aquaculture costs without compromising the health of marine organisms. Current research in this area is focused on fishmeal substitution, such as using soybean meal instead of fishmeal [8]. However, a high-lipid diet can reduce overall feed costs [9]. As an energy feedstuff, lipids can promote fish growth and conserve protein, making a high-lipid diet favored by the aquaculture industry [3,10]. The base feed for this experiment is a high-lipid diet, aligning with the contemporary theme of aquatic feed industry development.

Despite the advantages of a high-lipid diet in promoting growth and increasing protein efficiency, their disadvantages should not be overlooked [11]. Suo et al. demonstrated that a high-lipid diet (with a lipid level of 16.42%) could indeed promote growth and development [10]. However, they also observed a reduction in the lipid metabolic capacity of the liver, an increase in the fat content of fish muscle and the presence of fatty liver indicators in liver oil-red sections [10]. Therefore, there is an urgent need to find a suitable additive to address the shortcomings of high-lipid diets, which is currently a prominent research topic.

α-lipoic acid is an antioxidant and plays a role in the formation of various types of dehydrogenases involved in energy metabolism [12]. Its ability to regenerate allows it to provide continuous protection to organisms [13]. Adding α-lipoic acid to the diets of Nile tilapia (*Oreochromis niloticus*) and crucian carp (*Carassius auratus*) can enhance their weight gain rate and specific growth rate [14,15]. However, excessive amounts may adversely affect palatability [14]. Meanwhile, the antioxidant properties of α-lipoic acid have been demonstrated in various species, including wrinkled disc abalone [16,17].

The hybrid grouper (*Epinephelus fuscoguttatus* ♀ × *E. lanceolatus* ♂), a cross between the brown-spotted grouper and the saddle-banded grouper, is a carnivorous fish known for its unique meaty flavor [18]. It is popular among consumers and favored by farmers due to its superior growth rate, disease resistance and higher return on farm investment [19]. Positive feedback from both farmers and consumers has contributed to the production of pearl gentian grouper in China’s aquaculture, reaching up to 205,816 tonnes in 2022, making it one of the top three fish species in marine fish farming production [20]. Therefore, this study combines a high-lipid diet with an antioxidant (alpha-lipoic acid) to assess the advantages and disadvantages of the feed in terms of growth performance, liver antioxidation and intestinal health. This aims to provide reference data for the high-density aquaculture of hybrid groupers and enrich the nutritional experimental database for groupers.

## 2. Materials and Methods

### 2.1. Experiment Diets

According to the nutritional requirements of hybrid groupers, using fish meal, wheat gluten and casein as the main protein sources, and fish oil, corn oil and lecithin as the main oil sources, six iso-protein and iso-lipid diets with α-lipoic acid at the levels of 0, 0.04, 0.08, 0.12, 0.16 and 0.20% were made, named S1, S2, S3, S4, S5 and S6, respectively. We obtained the feed ingredients from Zhanjiang Yuehai Feed Co. (Zhanjiang, China). The ingredients were initially crushed and then sieved manually through a 60-mesh sieve. Following the feed formula (Table 1), precise calculations were performed, and the necessary components were carefully weighed and blended. We utilized a step-by-step expansion method for mixing the raw materials, starting with the lesser proportion of raw materials and eventually incorporating them using a V-mixer [21]. Subsequently, fish oil, corn oil and lecithin were introduced, thoroughly mixed, and filtered through a 40-mesh sieve. The oiled ingredients were then combined with water during mixing (at a ratio of 30% per kilogram of diet) and extruded in a twin-screw extruder (F–26, South China University of Technology, Guangzhou, Guangdong, China) before being air-dried naturally. Finally, the air-dried feed was packaged in plastic sealing bags and stored in a freezer at −20 °C.

### 2.2. Fish and Feeding Trial

The fish were procured from a farm in Zhanjiang (Guangdong Province, China) and then transported to the experimental aquaculture base of Guangdong Ocean University on Donghai Island (Zhanjiang, China). Groupers were temporarily housed in concrete ponds (5 m × 4 m × 1.8 m) to acclimate to their new environment and were fed commercial feed (50% protein level, 10% lipid level, Haida Aquatic Diet Co., Ltd., Zhanjiang, China) two times daily for one week. By the onset of the trial, the tanks underwent sterilization, and 540 fish, each weighing 8.97 ± 0.01 g, were randomly distributed within the tanks as soon as the culture facilities were prepared. The experimental setup included 6 groups, with 3 replicates in each group, totaling 18 tanks. Feeding took place at 8:00 a.m. and 4:00 p.m. Satiation feeding was implemented, and daily feed intake was meticulously recorded. After one hour of feeding, check each tank, remove residual feed in time, dry and record the weight. Any feces that may have accumulated at the tank bottoms were promptly removed at 2 h after feeding. Furthermore, 70% of the water in each tank was replaced daily. The daily water temperature, salinity and dissolved oxygen levels were measured, maintaining the following parameters: 27–31 °C, 26–28, and >7 mg/L, respectively, using a PTF-001B multi-parameter water quality detector (WBD Biotechnology Co., Ltd., Shanghai, China).

### 2.3. Sample Collection

After eight weeks of feeding experiments, we proceeded with sample collection. Feeding was halted the day before sampling. Fish were carefully removed from the tanks, weighed, and counted to assess growth performance, among other indexes. The experimental fish were then transferred to a sampling workstation located on ice to maintain optimal conditions. Blood samples were collected from six fish by gently inserting a 2.5 mL syringe into the caudal fin and transferring the blood to 1.5 mL centrifuge tubes. Four fish were delicately dissected to isolate the visceral mass, intestines and livers. A portion of the liver was transferred to a 2 mL cryotube for enzyme activity testing, while another portion was cut into small samples resembling soybeans and placed in RNA later for preservation. Finally, three fish underwent similar dissection procedures; a minor incision was made with a scalpel, and the liver samples were removed and preserved in formaldehyde solution for liver histology.

### 2.4. Methods of Analyses

#### 2.4.1. Growth Performance Formula

Weight gain rate (WGR, %) = 100 × [final body weight (FBW) − initial body weight (IBW)]/IBW; Specific growth rate (SGR, %/d) = 100 × [ln (FBW) − ln (IBW)]/days of the experiment. Survival rate (SR, %) = 100% × (total number of fish at termination/total number of fish stocked) Feed conversion ratio (FCR) = total dry feed intake/total weight gain.

The diets were analyzed according to the method of the Association of Official Analytical Chemists (AOAC) [22]. The moisture content was measured by drying at 105 °C to a constant weight, and ash content was determined by combustion at 550 °C for 6 h. The crude protein content was determined by the Kjeldahl method. The crude lipid content was determined by the Soxhlet extraction method [9].

#### 2.4.2. Measurement of Enzyme Activities

The activities of catalase (CAT), superoxide dismutase (SOD), glutathione peroxidase (GSH-Px), alkaline phosphatase (AKP), acid phosphatase (ACP), lysozyme (LYS), aspartate transaminase (AST), alanine transaminase (ALT) and the content of malondialdehyde (MDA), reactive oxygen species (ROS), and immunoglobulin M (IgM) were analyzed using commercial ELISA kits (Shanghai Enzyme-linked Biotechnology Co., Ltd., Shanghai, China) Total antioxidant capacity (T-AOC) in the serum and liver checked by the kit (DPPH method, Shanghai Enzyme-linked Biotechnology Co., Ltd., Shanghai, China). Liver samples were weighed and homogenized on ice with saline (1:9) (EasyWell series JY98-IIIN model cell crusher) after homogenization (TGL16 M Benchtop High-Speed Freezing Centrifuge by Shanghai Lu Xiang Yi Centrifuge Instruments Co., Shanghai, China.) at 3500 rpm for 15 min, extract the supernatant. All biochemical parameters were determined by Rayto, RT-6100 enzyme-linked immunosorbent assay. All index measures were carried out in strict accordance with the kit instructions, following a previously described method by Liu et al. [21].

#### 2.4.3. Hepatic Histological Structures

Specific production steps of sectioning included ethanol dehydration, embedding, sectioning, dewaxing and staining (85% ethanol for 5 min; 95% ethanol for 5 min; finally, stain sections with Eosin dye for min), rinsing with pure water and sealing with neutral resin. Liver sections were observed and measured by an inverted fluorescence microscope (Nikon Eclipse Ti-E).

#### 2.4.4. Analysis of Antioxidant and Immune-Related Gene Expression in Liver

One milliliter of Tranzol UP (TransGen Biotech, Beijing, China) was added to the samples, and total RNA was extracted according to the manufacturer’s protocol, and the quantity and quality of the isolated RNA was determined by a NanoDrop 2000 spectrophotometer (Gene Company Limited, Guangzhou, China) and 1% agarose gel electrophoresis at 260 nm and 280 nm, respectively. The first strand of cDNA was extracted using Evo M-MLV Kit AG11728 (Changsha, Hunan, China) and synthesized according to the manufacturer’s instructions. cDNA was stored at −20 °C for real-time quantitative polymerase chain reaction (RT-qPCR). RT-qPCR assays were performed using SYBR*^®^* Green Pro Taq HS qPCR (AG11702) and Roche Fluorescence quantification machines (Light Cycler 480II, Rotkreuz, Switzerland). The PCR conditions were set using a thermal programmer at 95 °C for 30 s, 40 cycles of 95 °C for 5 s and 60 °C for 34 s. Each sample was run in triplicate. RT-qPCR primers (Table 2) were designed based on published grouper sequences, and relative expression was calculated using the 2^−ΔΔCt^ method [23].

### 2.5. Statistical Analysis

Results are presented as “means ± standard error (SEM)”. Before performing a one-way analysis of variance (ANOVA), all data were tested for normality distribution (Kolmogorov–Smirnov test) and homogeneity of variances (Levene’s test), followed by Duncan’s multiple range tests. A *p*-value < 0.05 was considered significant. All statistical analyses were performed using SPSS version 20.0 ((SPSS Inc., Michigan Avenue, Chicago, IL, USA)) for Windows.

## 3. Results

### 3.1. Growth Performance

**Figure 1:**
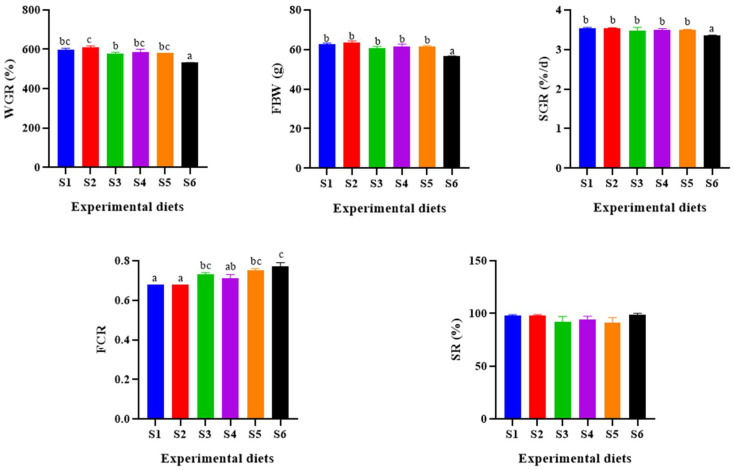
Effect of α-lipoic acid levels on growth performance of hybrid grouper. WGR, weight gain rate; FBW, final body weight; SGR, specific growth rate; FCR, feed conversion ratio; SR, survival rate. S1, control group. S2, 0.04% α-lipoic acid supplement group; S3, 0.08% α-lipoic acid supplement group; S4, 0.12% α-lipoic acid supplement group; S5, 0.16% α-lipoic acid supplement group; S6, 0.2% α-lipoic acid supplement group. Notes: Different letters assigned to the bars represent significant differences (*p* < 0.05).

The growth performance data are presented in Figure 1, providing insights into the differences among various groups. Notably, the FBW and WGR of the S6 group were significantly lower compared to the other groups (*p* < 0.05), while the S2 group exhibited the highest FBW, WGR and SGR values. However, these values did not significantly differ from those of the S1 group (*p* > 0.05). Furthermore, the FCR values for the S3, S5 and S6 groups were notably higher than those for the S1 and S2 groups, with the S6 group having the highest FCR value (*p* < 0.05). It is worth mentioning that the dietary levels of α-lipoic acid had no significant impact on SR (*p* > 0.05).

### 3.2. Serum Antioxidant Indexes

As depicted in Table 3, α-lipoic acid significantly enhanced the activities of antioxidant enzymes, including serum CAT, SOD, and GSH-Px in hybrid grouper. These enhancements were most pronounced in the S5 group, with all three enzymes reaching their maximum levels (*p* < 0.05). Notably, no significant change was observed in T-AOC.

### 3.3. Liver Antioxidant and Immune Indexes

As shown in Table 4, α-lipoic acid resulted in a significant reduction of ROS and MDA levels in the liver of hybrid groupers. In addition, liver CAT, SOD enzymes activities and T-AOC content showed a significant increase with increasing α-lipoic acid levels, reaching their peak values in the S5 group (*p* < 0.05). Furthermore, the enzyme activities of GSH-Px and AKP were significantly higher in all groups except the S3 group than in the S1 group (*p* < 0.05). Conversely, liver AST, ALT, ACP enzyme activities and IgM content remained largely unchanged despite variations in α-lipoic acid concentration (*p* > 0.05).

### 3.4. Liver Histology

**Figure 2:**
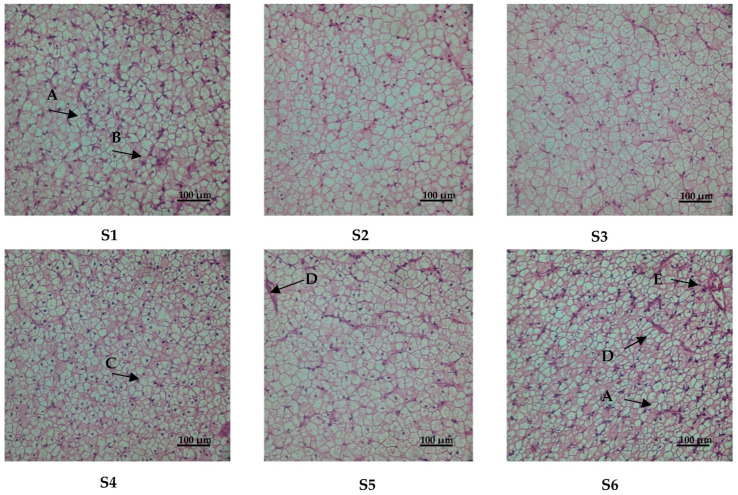
Effect of α-lipoic acid levels on the histological structure of liver (HE 400×). Notes: A, karyopyknosis; B, fat vacuolization; C, hepatocellular vacuolation; D, punctate necrosis; E, inflammation. S1, control group. S2, 0.04% α-lipoic acid supplement group; S3, 0.08% α-lipoic acid supplement group; S4, 0.12% α-lipoic acid supplement group; S5, 0.16% α-lipoic acid supplement group; S6, 0.2% α-lipoic acid supplement group.

The results of HE staining of the liver are shown in Figure 2. The hepatocytes of the S2 and S3 groups had complete hepatic lobules and clear cell outlines. In group S1, we observed karyopyknosis and fat vacuolization. In group S4, hepatocellular vacuolation was observed. Hepatocytes in the S5 group show punctate necrosis. In group S6, there was karyopyknosis, punctate necrosis and inflammation.

### 3.5. Antioxidant and Immune-Related Gene Expression in Liver

**Figure 3:**
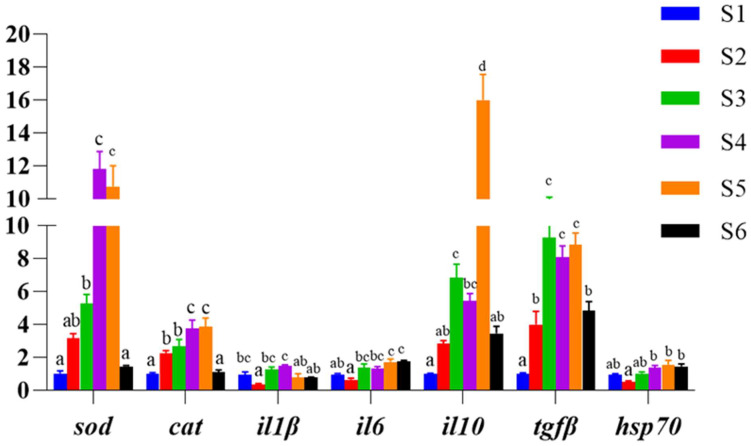
Effect of α-lipoic acid levels on relative gene expression of hepatic antioxidant-related and immune-related in hybrid grouper. Values are means ± SE (*n* = 3). S1, control group. S2, 0.04% α-lipoic acid supplement group; S3, 0.08% α-lipoic acid supplement group; S4, 0.12% α-lipoic acid supplement group; S5, 0.16% α-lipoic acid supplement group; S6, 0.2% α-lipoic acid supplement group. Notes: Different letters assigned to the bars represent significant differences (*p* < 0.05).

As shown in Figure 3, the expression of antioxidant genes superoxide dismutase (sod) and catalase (cat) (*p* < 0.05) was significantly upregulated in the liver except for the S6 group, and the expression of pro-inflammatory cytokine interleukin 1β (il1β) gene was significantly inhibited in the S2 group (*p* < 0.05), while the expression levels of interleukin 6 (il6) gene mRNA were significantly upregulated in the S5 and S6 groups (*p* < 0.05). The expression of the anti-inflammatory factors interleukin 10 (il10) and transforming growth factor β (tgfβ) showed a significant upward and then downward trend, and the expression level of il10 in all groups was significantly higher than that in the control group S1, except for the S2 and S6 groups, while the expression of tgfβ in all groups was significantly higher than that in the S1 group (*p* < 0.05). The expression level of the heat shock protein 70 (hsp70) was the lowest in the S2 group, but there was no significant difference between the treatment groups and the S1 group (*p* > 0.05).

## 4. Discussion

In our study, the parameters FBW (final body weight), WGR (weight gain rate) and SGR (specific growth rate) exhibited an increasing trend followed by a decrease with escalating levels of dietary α-lipoic acid supplementation. WGR reached their peak in group S2, showing no significant difference from the control group. However, FBW, WGR and SGR were significantly reduced at 2000 mg/kg alpha lipoic acid levels in the S6 group compared to the control group. Additionally, FCR (feed conversion ratio) in the S3, S5 and S6 groups significantly increased with higher α-lipoic acid levels. This phenomenon could be attributed to the reduced appetite and feeding behavior in fish due to the high concentration of α-lipoic acid, resulting in a significant decline in their overall growth performance. These findings align with previous studies, such as the research on *Trachinotus marginatus*, where α-lipoic acid inclusion at 316.4–524 mg/kg significantly improved WGR. However, at higher levels (890 and 1367 mg/kg), WGR diminished significantly, and FCR increased markedly [24]. Similarly, adding 1200 mg/kg α-lipoic acid to the diet of grass carp (*Ctenopharyngodon idella*) resulted in reduced feed intake and decreased FBW and WG [25]. Furthermore, in *Haliotis discus hannai*, WGR was enhanced when α-lipoic acid was included in the feed, peaking at 800 mg/kg, but declined considerably at 1600 and 3200 mg/kg [16]. In summary, the addition of α-lipoic acid to the diet positively influences animal growth within specific concentration ranges. However, excessive levels of α-lipoic acid can suppress appetite and food intake, ultimately hampering overall growth performance. These observations emphasize the importance of carefully regulating α-lipoic acid supplementation to optimize growth outcomes in fish.

The antioxidant defense mechanism of the body is a set of antioxidant enzymes, including T-AOC, CAT, SOD and GSH-Px, which protect the body from damage caused by reactive oxygen species [26,27]. CAT also plays an important role in the antioxidant defense of the body by converting H_2_O_2_ to O_2_ and water, thus protecting cells from damage caused by hydrogen peroxide [28,29]. In addition, glutathione peroxidase (GSH-Px), an important peroxidolytic enzyme widely present in the organism, reduces toxic peroxides to non-toxic hydroxyl compounds, thus protecting cell structure and function from peroxide interference and damage [29,30]. Excessive fat intake and deposition can aggravate the degree of oxidative damage in the organism [4,31]. The oxidative and antioxidant defense systems in animals are in a dynamic equilibrium under normal physiological conditions, and when the organism is stressed, the intracellular mitochondrial morphology is altered, leading to abnormal function and the production of ROS [32,33]. High levels of ROS tend to attack important intracellular biomolecules such as lipids, proteins and nucleic acids, triggering oxidative stress in the organism [34,35]. Malondialdehyde (MDA) is a product of the peroxidation of polyunsaturated fatty acids, and its level is a measure of the degree of oxidative stress [27,36,37]. In the present experiment, CAT, SOD and GSH-Px enzyme activities in serum and liver, as well as T-AOC in the livers of groupers, were significantly increased with increasing α-lipoic acid concentration, while ROS and MDA levels were significantly decreased. This suggests that α-lipoic acid as an additive can attenuate oxidative damage in the body by enhancing the activity of free radical scavenging enzymes and improving the antioxidant capacity of hybrid grouper. Similarly, a study in grass carp concluded that α-lipoic acid as a feed additive could modulate the antioxidant defense system of the fish, significantly increase the activity of antioxidant enzymes in the liver and serum, reduce MDA accumulation and attenuate the toxic effects of lipid peroxidation [38]. The same findings were also demonstrated in the Chinese mitten crab (*Eriocheir sinensis*) [39], carp (*Cyprinus carpio*) [38] and tilapia (*Oreochromis niloticus*) [12]. This also corresponds to the improvement of the liver structure. Alkaline phosphatase (AKP) plays a crucial role in the regulation of animal metabolic processes, helps to maintain a stable internal environment and organism health [40,41], and is associated with organism growth [42,43,44]. On the other hand, lysozyme (LYZ) has been identified as a significant defense factor for vertebrates against invading microorganisms [44,45,46]. LYZ is effective in lysing Gram-positive bacteria, killing Gram-negative bacteria and promoting phagocytosis, either by regulation or through the activation of polymorphonuclear leukocytes and macrophages [47,48,49]. In the course of our experiment, we observed that α-lipoic acid significantly increased the activities of AKP and LYZ enzymes in the liver. This suggests that the addition of α-lipoic acid may enhance the antimicrobial capacity. Additionally, a similar effect was noted when 600 mg/kg of α-lipoic acid was added to the feed of Nile tilapia, which led to a notable increase in the activity of LYZ in serum [15]. Likewise, the introduction of 351 mg/kg of α-lipoic acid significantly boosted LYZ activity and IgM levels in the serum of grass carp [50]. Collectively, these results indicate that α-lipoic acid has the potential to enhance an organism’s immunity.

The histological liver morphology, as determined through HE staining, serves as a critical indicator for evaluating the physiological well-being of the fish and identifying the presence or absence of liver lesions [51]. Previous research has established that high-lipid diets can induce liver damage, characterized by nucleus displacement, nucleolysis, loss of cell structure and cellular vacuolization [52,53]. Our examination of liver sections revealed partial damage in groups S1, S4, S5 and S6. In a prior study, we successfully mitigated similar liver pathologies by supplementing with VE [54], choline [3] and tea polyphenols [53]. In the current study, α-lipoic acid produced similar beneficial effects, which can likely be attributed to its role as an antioxidant. This role enhances the organism’s antioxidant capacity, effectively scavenging lipid peroxidation resulting from high-fat diets. This, in turn, safeguards the organism’s health and preserves normal liver metabolism.

The activity of antioxidant enzymes plays an important role in the antioxidant defense system, and their activity levels are regulated by antioxidant enzymes-related genes, such as *sod* and *cat*. In the present experiment, the mRNA expression levels of both *sod* and *cat* were significantly upregulated in the liver with increasing levels of α-lipoic acid addition, which is consistent with the trend of their enzyme activities, again confirming that α-lipoic acid can improve the antioxidant capacity of the organism. It was found that the upregulation of pro-inflammatory factors and the downregulation of anti-inflammatory cytokines leads to inflammatory responses in fish [38]. *il1β* is an important member of the il1 family, which is of interest due to its important role in inflammation-related diseases [55]. *il1β* has strong pro-inflammatory activity and induces a variety of pro-inflammatory mediators, such as cytokines and chemokines, and its local activation is central to mediating pro-inflammatory responses that lead to the activation of secondary inflammatory mediators (including *il6*) [53,56]; *il-10* and *tgfβ* are important anti-inflammatory factors that significantly reduce inflammation by inhibiting the expression of pro-inflammatory factors [15]. In the present study, the expression of the pro-inflammatory factor *il1β* was significantly downregulated in the S2 group, while the expression of *il6* was significantly upregulated in the S5 and S6 groups with increasing α-lipoic acid concentration. Conversely, the mRNA expression of both the anti-inflammatory factor *il10* and *tgfβ* was significantly upregulated, which may represent a feedback response of the organism to the upregulation of pro-inflammatory factors. Under normal physiological conditions, the expression level of *hsp70* is typically low, but it rapidly and significantly increases in response to external stimuli [11,57,58]. In this experiment, the *hsp70* expression level in all groups did not significantly differ from the S1 group, except in the S2 group, where it was significantly lower than in the S4, S5 and S6 groups. This could be attributed to the organism’s relatively high immunity when α-lipoic acid was added at 400 mg/kg. Currently, α-lipoic acid has been shown to enhance immune responses in mammals, with the addition of 900 mg/kg α-lipoic acid to the diet significantly elevating serum levels of interleukin 2 (*il2*) and decreasing levels of inflammatory cytokines, such as *il1β*, *il6*, and *tnfα* in fattening pigs [59]. Similarly, the addition of 100 mg/kg α-lipoic acid to feed significantly down-regulated the expression level of *tnfα* in rat liver [60]. In aquatic animals, the addition of appropriate amounts of α-lipoic acid to feed significantly up-regulated the levels of inflammatory cytokines such as *tgfβ1*, growth transformation factor β2 (*tgfβ2*), white fine *il10* and interleukin 11 (*il11*) in the head, kidney and spleen of grass carp [25]. This is also consistent with the results of this experiment. In conclusion, the addition of α-lipoic acid to a high-lipid diet can improve the immunity of the organism by regulating the expression of non-specific related genes and altering enzyme activities.

## 5. Conclusions

In this experiment, 2000 mg/kg of α-lipoic acid can inhibit grouper growth. At the appropriate level of supplementation (400–800 mg), it can improve the shape of liver cells in the liver, but excess (1600–2000 mg) can lead to inflammation of liver cells and punctate necrosis. In the 400–1600 mg/kg α-lipoic acid groups, antioxidant activity, immunoenzyme activity and expression of antioxidant and immune-related genes could be improved to a certain extent. In summary, the recommended supplemental dose of alpha lipoic acid is 400–800 mg/kg.

## Figures and Tables

**Table 1:** Ingredient composition and nutrient content of the test diets (% dry matter).

Ingredient	S1	S2	S3	S4	S5	S6
Fish meal	43	43	43	43	43	43
Wheat gluten	10	10	10	10	10	10
Casein	12	12	12	12	12	12
Wheat flour	17	17	17	17	17	17
Soybean lecithin	1.5	1.5	1.5	1.5	1.5	1.5
Fish oil	5	5	5	5	5	5
Corn oil	7	7	7	7	7	7
Gelatinized starch	0.7	0.66	0.62	0.58	0.54	0.5
Compound premix ^a^	1	1	1	1	1	1
Vitamin C	0.05	0.05	0.05	0.05	0.05	0.05
Choline chloride	0.5	0.5	0.5	0.5	0.5	0.5
Ca(H_2_PO_4_)_2_	1.5	1.5	1.5	1.5	1.5	1.5
Antioxidant ^b^	0.1	0.1	0.1	0.1	0.1	0.1
Attractant ^c^	0.15	0.15	0.15	0.15	0.15	0.15
CMC ^d^	0.5	0.5	0.5	0.5	0.5	0.5
α-lipoic acid	0	0.04	0.08	0.12	0.16	0.2
Total	100	100	100	100	100	100
Proximate composition ^e^						
Moisture	9.23	10.09	10.95	9.8	9.38	10.44
Crude protein	50.62	51.18	51.55	50.94	50.84	50.06
Crude lipid	16.38	15.72	16.14	15.75	16.16	16.2
Ash	12.74	12.15	12.09	12.86	12.45	12.79

^a^ Compound premix was obtained from Qingdao Master Biotechnology Co, Ltd. (Qingdao, China). ^b^ Antioxidant: ethoxyquin. ^c^ Attractant composition:taurine:glycine:betaine = 1:3:3. ^d^ Carboxymethylcellulose sodium. ^e^ Measured value.

**Table 2:** Primers of RT-Qpcr.

Genes	5=/3 = Forward Primer	5=/3 = Reverse Primer	Amplicon	E-Value %	Genbank No.
*β-actin*	ACTGCTGCCTCCTCTTCATC	ACCGCAAGACTCCATACCAA	135	93.71	KU746361.1
*sod*	TGGAAACACCTTTCCCCCAC	CTGACAGGGTAAAGCATGGC	120	91.41	AY735008.1
*cat*	CGCGGGAAGCAAAGATTCAG	CCGCAGTTTCCAGTGTGTTG	194	104.32	KT884509.1
*il6*	AGGAAGTCTGGCTGTCAGGA	GCCCTGAGGCCTTCAAGATT	250	95.06	JN806222.1
*tgf* *β*	CGATGTCACTGACGCCCTGC	AGCCGCGGTCATCACTTATC	107	90.00	GQ205390.1
*il1β*	CGACATGGTGCGGTTTC	TCTGTAGCGGCTGGTGG	151	91.95	EF582837.1
*il10*	ACACAGCGCTGCTAGACGAG	GGGCAGCACCGTGTTCAGAT	104	91.86	KJ741852.1
*hsp70*	CTTGCAAGAAGTGGCCAACA	AAAGCCATCTTCCTGCCTTGT	131	94.03	EU816600.1

Notes: *sod*, superoxide dismutase; *cat*, catalase; *il6*, interleukin 6; *tgfβ*, transforming growth factor β; *il-1β*, interleukin 1β; *il10*, interleukin 10; *hsp70*, heat shock protein 70.

**Table 3:** Effect of α-lipoic acid levels on serum antioxidant parameters.

Group	CAT (U/mL)	T-AOC (U/mL)	SOD (U/mL)	GSH-Px (U/L)
S1	25.21 ± 2.06 ^a^	13.13 ± 0.96	62.06 ± 3.33 ^a^	62.89 ± 3.53 ^a^
S2	29.02 ± 2.01 ^ab^	12.47 ± 0.34	83.4 ± 2.94 ^b^	73.79 ± 8.01 ^ab^
S3	30.07 ± 1.91 ^ab^	13.01 ± 0.54	87.88 ± 4.63 ^b^	75.53 ± 4.56 ^abc^
S4	34.51 ± 3.41 ^b^	14.21 ± 1.67	97.16 ± 6.16 ^b^	92.41 ± 4.99 ^cd^
S5	44.51 ± 2.95 ^c^	15.61 ± 1.26	117.03 ± 7.68 ^c^	102.04 ± 5.53 ^d^
S6	36.51 ± 1.53 ^b^	14.46 ± 1.04	90.71 ± 4.41 ^b^	82.18 ± 6.25 ^bc^

Notes: Values in the table are means ± SEM (*n* = 3); Values in the same column with the same superscript letter or absence of superscripts are not significantly different (*p* > 0.05). S1, control group. S2, 0.04% α-lipoic acid supplement group; S3, 0.08% α-lipoic acid supplement group; S4, 0.12% α-lipoic acid supplement group; S5, 0.16% α-lipoic acid supplement group; S6, 0.2% α-lipoic acid supplement group.

**Table 4:** Effects of α-lipoic acid levels on liver antioxidant and immunity parameters.

Group	S1	S2	S3	S4	S5	S6
ROS (U/mg.pro)	352.65 ± 4.44 ^c^	370.31 ± 9.79 ^c^	292.77 ± 27.48 ^b^	225.17 ± 13.39 ^a^	253.06 ± 10.86 ^ab^	269.87 ± 5.34 ^ab^
MDA (nmol/mg.pro)	15.98 ± 0.81 ^c^	16.03 ± 1.34 ^c^	13.29 ± 0.82 ^b^	9.65 ± 0.68 ^a^	7.84 ± 0.26 ^a^	8.93 ± 0.4 ^a^
CAT (U/mg.pro)	38.44 ± 5.69 ^a^	60.74 ± 6.29 ^b^	52.33 ± 4.51 ^ab^	62.3 ± 3.32 ^b^	79.08 ± 3.28 ^c^	56.45 ± 4.45 ^b^
T-AOC (U/mg.pro)	9.55 ± 0.61 ^a^	15.98 ± 1.19 ^b^	15.55 ± 1.62 ^b^	15.67 ± 1.17 ^b^	21.28 ± 1.01 ^c^	16.19 ± 0.67 ^b^
SOD (U/mg.pro)	118.27 ± 2.52 ^a^	174.78 ± 1.01 ^b^	168.09 ± 22.81 ^b^	166.15 ± 10.66 ^b^	179.57 ± 2.41 ^b^	163.09 ± 16.62 ^b^
GSH-Px (mU/mg.pro)	125.57 ± 11.45 ^a^	178.02 ± 18.18 ^b^	150.48 ± 6.5 ^ab^	165.11 ± 15.59 ^b^	178 ± 8.01 ^b^	168.58 ± 4.94 ^b^
AKP (mIU/mg.pro)	11.89 ± 1.95 ^a^	20.32 ± 2.2 ^b^	16.14 ± 0.8 ^ab^	21.02 ± 0.27 ^b^	17.88 ± 1.45 ^b^	19.13 ± 1.19 ^b^
ACP (mU/mg.pro)	14.80 ± 1.22	12.65 ± 1.28	12.6 ± 1.61	14.75 ± 0.17	14.18 ± 1.39	11.9 ± 1.05
AST (mU/mg.pro)	20.11 ± 1.28	17.18 ± 1.57	17.53 ± 2.54	17.84 ± 0.24	15.03 ± 2.62	16.31 ± 1.07
ALT (mU/mg.pro)	9.54 ± 0.6	10.86 ± 1.45	9.24 ± 1.49	8.85 ± 1.46	7.72 ± 1.01	9.04 ± 0.47
IgM (ug/mg.pro)	49.58 ± 3.52	54.84 ± 4.36	51.17 ± 1.85	52.17 ± 4.7	56.94 ± 5.24	51.14 ± 2.31
LYZ (mU/mg.pro)	6.49 ± 0.62 ^a^	9.87 ± 0.34 ^c^	8.12 ± 0.53 ^b^	9.00 ± 0.56 ^bc^	9.51 ± 0.56 ^bc^	9.24 ± 0.23 ^bc^

Notes: Values in the table are means ± SEM (*n* = 3); Values in the same column with the same superscript letter or absence of superscripts are not significantly different (*p* > 0.05). S1, control group. S2, 0.04% α-lipoic acid supplement group; S3, 0.08% α-lipoic acid supplement group; S4, 0.12% α-lipoic acid supplement group; S5, 0.16% α-lipoic acid supplement group; S6, 0.2% α-lipoic acid supplement group.

## Data Availability

The data that support the findings of this study are available on request from the corresponding author. The data are not publicly available due to privacy or ethical restrictions.
